# Data on autophagy markers in human macrophages exposed to oxLDL and growth differentiation factor-15

**DOI:** 10.1016/j.dib.2019.103728

**Published:** 2019-03-06

**Authors:** Kathrin Ackermann, Gabriel A. Bonaterra, Ralf Kinscherf, Anja Schwarz

**Affiliations:** Institute for Anatomy and Cell Biology, Department of Medical Cell Biology, Philipps-University of Marburg, 35032 Marburg, Germany

## Abstract

Growth differentiation factor-15 (GDF-15) is a member of the TGF-β superfamily, identical to MΦ-inhibitory cytokine-1 (MIC-1). GDF-15 is associated with e.g. cardiovascular disease, inflammation and development of atherosclerosis and is highly expressed in macrophages (MΦ) of atherosclerotic lesions. Moreover, there exists an indication for the involvement of oxidized-low density lipoprotein (oxLDL) uptake and autophagic processes by MΦ regarding arteriosclerotic progression. Thus, we were interested to investigate a potential regulatory effect of GDF-15 on autophagy signaling pathway in human MΦ during foam cell formation. Here, we present western blot data of ATG5, ATG12/ATG5-complex and p62 regarding the GDF-15 concentration. For further interpretation of the data presented in this article, please see the research article “Growth differentiation factor-15 regulates oxLDL-induced lipid homeostasis and autophagy in human macrophages” [1].

**Table:** Specifications table

Subject area	*Biology*
More specific subject area	*Autophagy*
Type of data	*Graph, figure*
How data was acquired	*Western blot;* The intensity of the specific western blot bands was quantified using the software ImageJ from the National Institutes of Health (Bethesda, USA).
Data format	*Analyzed*
Experimental factors	*THP-1 cells were cultured in RPMI-1640 medium (Capricorn Scientific GmbH, Ebsdorfergrund, Germany) supplemented with penicillin and streptomycin (Capricorn Scientific GmbH,) and 10% fetal bovine serum (Capricorn Scientific GmbH,). Cells were cultured at 37°C in a 5% C02 environment. RPMI 1640 medium supplemented with* 100 nM *Phorbol 12-mystriate 13-acetate [PMA, (Sigma-Aldrich Chemie GmbH Munich, Germany)] was used (72h) to THP-1 cells to induce monocyte differentiation into MΦ. Transfection of THP-1 MФ with* 50 nM *small interfering RNA (siRNA) for GDF-15 (FlexiTube GeneSolution GS9518, QIAGEN GmbH, Hilden, Germany) and with negative siRNA (nsiGDF-15) (AllStars Negative Control, QIAGEN GmbH) was performed using HiPerfect Transfection Reagent (QIAGEN GmbH) following the manufacturer's instructions. After transfection, cells were treated with* 50 μg/ml *oxLDL to induce foam cell formation or left untreated (medium) for 4h. THP-1 MΦ were treated with human rGDF-15 [*20ng/ml*, 1.0-2.*0 μg/ml *rGDF-15 (ProVitro AG, Berlin, Germany)] or co-incubated with oxLDL + rGDF-15 for 4h.*
Experimental features	The peroxidase reaction was visualized by AceGlow chemiluminescence substrate (PEQLAB GmbH, Erlangen, Germany) and documented by the Fusion-SL Advance™ imaging system (PEQLAB GmbH)
Data source location	*Philipps-University Marburg, Marburg, Germany*
Data accessibility	*All data are presented in this article.*
Related research article	K. Ackermann, G.A. Bonaterra, R. Kinscherf, A. Schwarz (2019) Growth differentiation factor-15 regulates oxLDL-induced lipid homeostasis and autophagy in human macrophages. Atherosclerosis 19:128–136. https://doi.org/10.1016/j.atherosclerosis.2018.12.009.

**Table:** 

**Value of the data** • The data show the effect of several rGDF-15-concentrations on autophagy markers in THP-1 MΦ. • These data may be relevant for (i) other researchers using rGDF-15 in their experiments and (ii) for further research that focuses on autophagy-signaling dependent on rGDF-15.

## 1 Data

**Fig. 1:**
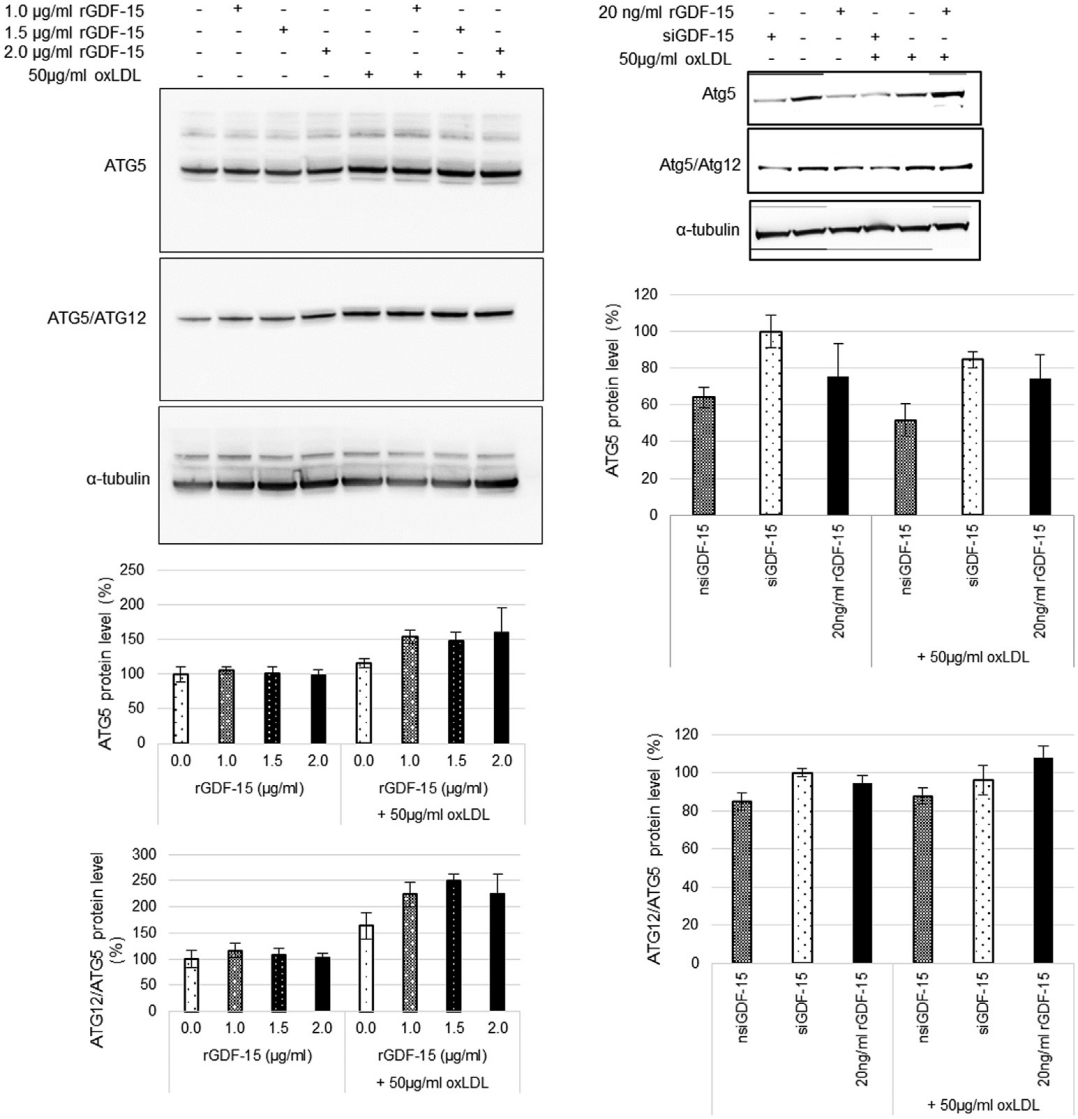
Data of GDF-15-effect on the expression of autophagy-relevant proteins ATG5 and ATG12/ATG5-complex in *THP-1 MΦ*. Percentage of Atg5 and ATG12/ATG5-complex protein levels in *THP-1 MΦ*. Expression was normalized against α-tubulin and quantified by ImageJ. Representative images of Western blot results for ATG5, ATG12/ATG5-complex and α-tubulin. Data are presented as mean ± SEM (four – six independent experiments were performed).

**Fig. 2:**
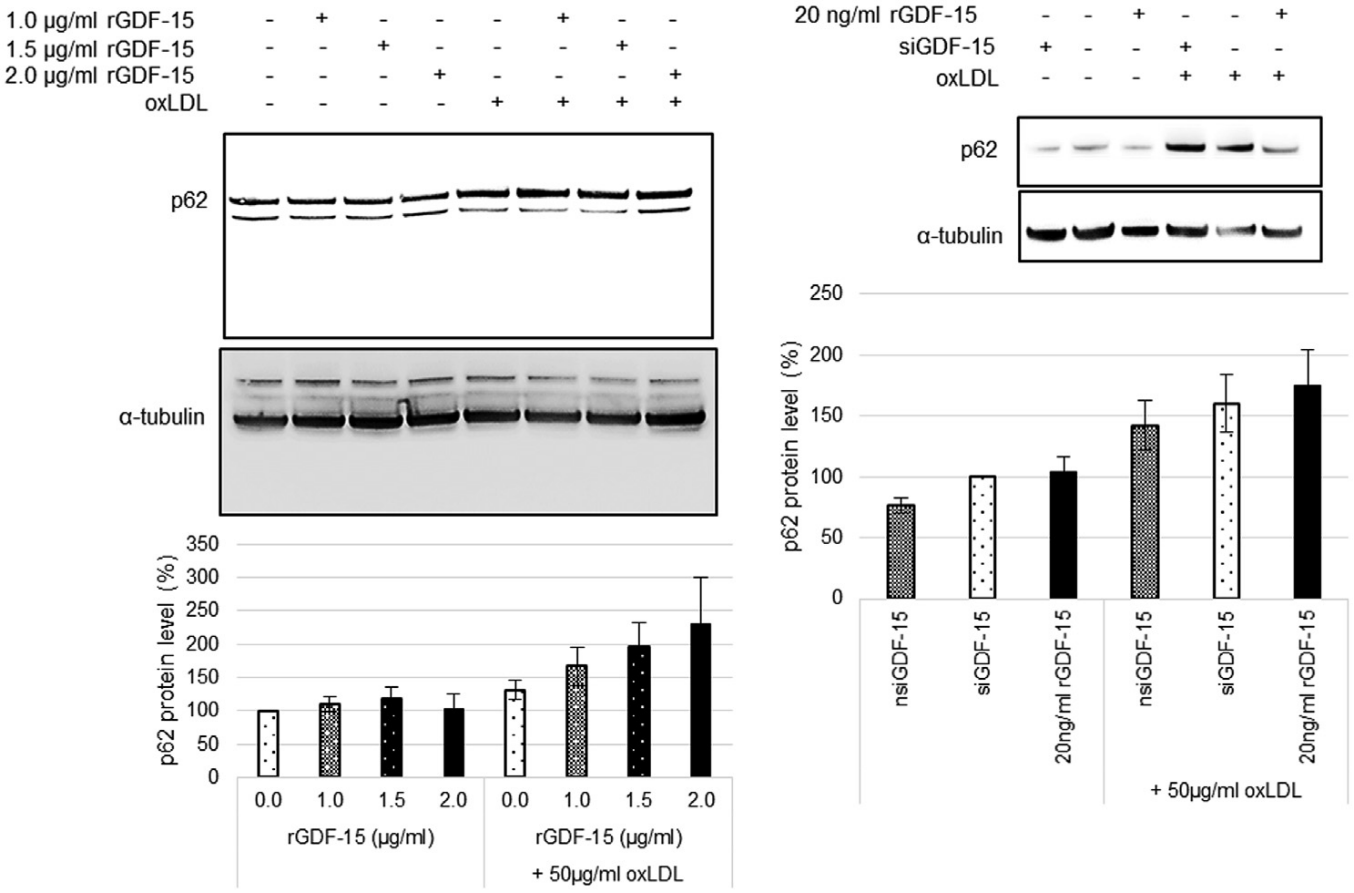
Data of GDF-15-effect on the expression of p62 in *THP-1 MΦ*. Percentage of p62 protein level in *THP-1 MΦ*. Expression was normalized against α-tubulin and quantified by ImageJ. Representative images of Western blot results for p62 and α-tubulin. Data are presented as mean ± SEM (six - nine independent experiments were performed).

This Data in Brief present a data set of autophagy-relevant proteins/complexes (ATG5, ATG12/ATG5 and p62) dependent from rGDF-15 and oxLDL in human THP-1 MΦ. The normalized western blot data of the THP-1 MΦ are presented in Fig. 1 and Fig. 2. The intensity of the specific western blot bands was quantified using the software ImageJ from the National Institutes of Health (Bethesda, USA) and normalized against α-tubulin. Treatment of THP-1 MΦ with rGDF-15 alone for 4h did not affect the ATG5, ATG12/ATG5-complex protein and p62 (Fig. 1, Fig. 2) independent of the rGDF-15 concentration. GDF-15 silencing (siGDF-15) decreased ATG5, ATG12/ATG5-complex and p62 (Fig. 1, Fig. 2). Data of THP-1 MΦ co-incubated with oxLDL/rGDF-15 (4h) show an increased ATG5-, ATG12/ATG5-complex and p62-protein level, whereas 2.0 μg/ml rGDF-15 did not amplify the effect of 1.0 μg/ml and 1.5 μg/ml rGDF-15. The low concentration of 20 ng/ml rGDF-15 had no effect on ATG5-, ATG12/ATG5-complex and p62-protein level in untreated or oxLDL-treated THP-1 MΦ.

## 2 Experimental design, materials and methods

### 2.1 LDL-oxidation

Native (n)LDL (RayBiotech, GA, USA) oxidation was performed as previously described by Galle and Wanner [2] and Steinbrecher [3]. The nLDL was suspended in endotoxin-free phosphate-buffered saline (PBS) without Ca^2+^, Mg^2+^ (LONZA, Ratingen, Germany) to a final concentration of 1 mg protein/ml and dialyzed using Slide-A-Lyzer Dialysis Cassettes 7K MWCO (Thermo Fisher Scientific Inc., Rockford, USA). OxLDL was obtained by oxidizing nLDL using CuSO_4_ (50 μM in PBS Ca^2+^, Mg^2+^ free, 24 h, RT). Different methods verified the grade of oxidation: (1) - Trinitrobenzene sulfonic acid (TNBSA, Thermo Fisher Scientific Inc., Rockford, USA), which measures free amino groups [4]. (2) - Relative electrophoretic mobility (REM) by agarose gel electrophoresis and visualized by staining with Coomassie Blue [5], and (3) - by spectrophotometric analysis (absorbance spectrum between 400 and 700nm [2]. OxLDL was used by an increased REM of 17.5% ± 3.34% compared to nLDL, an increased percentage of blocked amino groups (40.4% ± 0.65% compared to LDL) and by the disappearance of the nLDL characteristic absorption peaks at 460 and 485 nm [see supplementary data Ref. [1]].

### 2.2 Cell culture, transfection and gene silencing

The human leukemic monocyte cell line *THP-1* (Leibniz Institute DSMZ, Braunschweig, Germany) was used. Originally derived from the blood of a one-year old boy with acute monocytic leukemia, these cells are frequently used as a model of monocyte/MΦ cell lineage [6]. *THP-1* cells were cultured in RPMI-1640 medium (Capricorn Scientific GmbH, Ebsdorfergrund, Germany) supplemented with 10% fetal bovine serum (Capricorn Scientific GmbH)and 5% penicillin and streptomycin (Capricorn Scientific GmbH) at 37 °C in a 5% C0_2_ environment with a medium change every 2–3 days. All experiments were performed using cells at passage 9 and lower. To induce monocyte differentiation into MΦ, RPMI 1640 medium was supplemented with 100 nM Phorbol 12-mystriate 13-acetate [PMA, (Sigma-Aldrich Chemie GmbH Munich, Germany)] for 72h. Transfection of *THP-1 MФ* with 50 nM small interfering RNA (siRNA) for *GDF-15* (FlexiTube GeneSolution GS9518, QIAGEN GmbH, Hilden, Germany) and with negative siRNA (*nsiGDF-15*) (AllStars Negative Control, QIAGEN GmbH) was performed using HiPerfect Transfection Reagent (QIAGEN GmbH) following the manufacturer's instructions. AllStars Hs Cell Death Control siRNA (QIAGEN GmbH), a compound of highly potent siRNAs targeting ubiquitously expressed human genes that are essential for cell survival, was used as a positive control. Transfection efficiency was estimated by observing cells by light microscopy 48 h after transfection with the AllStars Hs Cell Death Control siRNA. After transfection, foam cell formation was induced by treating the cells with 50 μg/ml oxLDL for 4h. As control, cells were left untreated (medium) for the same amount of time. Because of the biological activity of rGDF-15 ED50 = 1.0–3.0 μg/ml (ProVitro, Germany), PMA-differentiated THP-1 MΦ were treated with 0.02–2.00 μg/ml human rGDF-15 [rGDF-15 (ProVitro AG, Berlin, Germany)] or co-incubated with oxLDL + rGDF-15 for 4h.

### 2.3 SDS-PAGE and western blot

After the treatments, PMA-differentiated *THP-1 MΦ* were washed in ice-cold PBS and lysed using radioimmunoprecipitation assay (RIPA) buffer pH 7.5 (Cell Signaling Technology, Frankfurt, Germany), containing protease/phosphatase inhibitor cocktail (Cell Signaling Technology). The Pierce BCA (bicinchoninic acid) Protein Assay (Thermo Scientific, Rockford, USA) was used to spectrophotometrically determine protein concentrations. Thereafter, proteins were loaded on NuPAGE^®^ Novex^®^ 4–12% Bis-Tris Gels, pre-cast polyacrylamide gels (Life Technologies GmbH, Darmstadt Germany), followed by the transfer onto a 0.45 μm nitrocellulose membrane (Millipore, Billerica, MA, USA). Primary Antibodies [see supplementary data Ref. [1]] were added in blocking buffer (5% fat-free milk) and incubated overnight at 4 °C. Membranes were incubated with enhanced ECL-anti-goat IgG-POD antibody, ECL-anti-mouse IgG-POD antibody or ECL-anti-rabbit IgG-POD antibody. AceGlow chemiluminescence substrate (PEQLAB GmbH, Erlangen, Germany) was added to visualize the peroxidase reaction and for documentation we used the Fusion-SL Advance™ imaging system (PEQLAB GmbH) according to the manual instructions. The software ImageJ from the National Institutes of Health (Bethesda, USA) was used to quantify the intensity of the specific western blot bands and α-tubulin.

### 2.4 Statistical analyses

Statistical analyses were performed using SigmaPlot 12 (Systat Software Inc., USA). After testing for normality (by Shapiro-Wilk), the unpaired Student's t-test or one-way analysis of variance (ANOVA) was used. Data are reported as mean ± standard error of the mean (S.E.M).
